# Case Report: Acute interstitial pneumonia as the initial presentation of Anti-EJ antisynthetase syndrome: a case of severe respiratory failure with rapid response to cyclophosphamide

**DOI:** 10.3389/fimmu.2026.1877724

**Published:** 2026-08-12

**Authors:** Katarzyna Królak-Nowak, Konrad Czchowski, Wiktoria Łoskot-Pawlik, Aleksandra Opinc-Rosiak, Joanna Makowska, Adam Antczak

**Affiliations:** 1 Department of General and Oncological Pulmonology, Medical University of Lodz, Lodz, Poland; 2 Department of Rheumatology, Medical University of Lodz, Lodz, Poland

**Keywords:** acute interstitial pneumonia, Anti-EJ antibodies, antisynthetase syndrome, cyclophosphamide, glycyl-tRNA synthetase, rapidly progressive interstitial lung disease

## Abstract

**Background:**

Antisynthetase syndrome (ASyS) associated with anti-EJ antibodies (directed against glycyl-tRNA synthetase) is increasingly recognized as an ILD-dominant phenotype with a propensity for rapidly progressive ILD (RP-ILD). Acute interstitial pneumonia (AIP)-like presentations may mimic severe infection, posing significant diagnostic challenges.

**Case presentation:**

A 55-year-old man with hypertension and a 20-pack-year smoking history presented with a fever (39°C) and rapidly progressive dyspnea. Laboratory findings revealed markedly elevated inflammatory markers. CT pulmonary angiogram demonstrated diffuse bilateral ground-glass opacities, consolidations, and interlobular septal thickening consistent with AIP-pattern. Despite broad-spectrum antibiotics, the patient developed refractory respiratory failure requiring mechanical ventilation, hemodynamic instability requiring vasopressors, and acute kidney injury. Bronchoscopy with transbronchial biopsy confirmed acute interstitial inflammation. After more than one week in the intensive care unit (ICU) without improvement, rheumatologic evaluation revealed positive for anti-EJ and anti-Th/To antibodies, supporting ASyS. High-dose of immunosuppressive drug were initiated, resulting in marked improvement in oxygenation, hemodynamics, and muscle strength within 48 hours. Follow-up CT at three weeks demonstrated significant regression of ground-glass opacities with residual findings consistent with nonspecific interstitial pneumonia/usual interstitial pneumonia (NSIP/UIP) overlap.

**Conclusion:**

ASyS should be considered in patients with severe pneumonia-like illness who fail to respond to appropriate antibiotic therapy. Anti-EJ antibodies are associated with AIP-like patterns and fulminant respiratory failure. Early serologic testing and prompt immunosuppression can be life-saving. The recently published 2024 Classification Criteria for Anti-Synthetase Syndrome (CLASS) provide a contemporary framework for the classification of ASyS, particularly in ILD-dominant presentations.

## Introduction

Antisynthetase syndrome (ASyS) is a heterogeneous autoimmune condition characterized by the presence of anti–aminoacyl tRNA synthetase antibodies, including anti-EJ antibodies directed against glycyl-tRNA synthetase, and a characteristic constellation of clinical features, most commonly interstitial lung disease (ILD), myositis, inflammatory arthritis, Raynaud phenomenon, fever and mechanic’s hands (1–4). Recognition of non-Jo-1 antibodies has markedly increased in recent years, revealing distinct serologic–clinical phenotypes with variable organ involvement and prognosis (2, 5, 6). Among these, anti-EJ antibodies are strongly associated with ILD-dominant disease, often presenting without overt myositis and with a tendency toward rapidly progressive ILD (RP-ILD) (7–10). Contemporary multicenter cohorts suggest that anti-EJ-positive ASyS frequently presents with ILD, frequently exhibiting AIP-like radiologic patterns, severe hypoxemia, and early respiratory failure, with rapidly progressive ILD independently associated with mortality (7–9, 11, 12).

Radiologic manifestations of ASyS-associated ILD span nonspecific interstitial pneumonia (NSIP), organizing pneumonia (OP), NSIP/OP overlap, usual interstitial pneumonia (UIP) and, less commonly, AIP (8, 10, 13, 14). AIP-like presentations are particularly relevant in anti-EJ disease, where fulminant respiratory deterioration may mimic severe infection (7–9, 11). Although rare, diffuse alveolar hemorrhage (DAH) has been increasingly recognized as a part of the pulmonary spectrum of idiopathic inflammatory myopathies (IIM) and ASyS (15–17).

Historically, classification systems such as the 2017 European Alliance of Associations for Rheumatology/American College of Rheumatology (EULAR/ACR) classification criteria for IIM did not recognize ASyS as a distinct entity (18). The recently published 2024 Classification Criteria for Anti-Synthetase Syndrome (CLASS) address this gap by integrating serologic markers with weighted clinical and organ-specific domains, allowing for more accurate classification of ASyS, particularly in patients presenting primarily with ILD and non-Jo-1 antibodies (19, 20).

We present a case of severe AIP-like respiratory failure as the initial manifestation of anti-EJ ASyS, characterized by profound hypoxemia, multiorgan dysfunction and rapid improvement after initiation of high-dose methylprednisolone and cyclophosphamide. This case highlights the diagnostic complexity of differentiating severe pneumonia from autoimmune ILD, underscores importance of early serologic testing and emphasizes the therapeutic benefit of prompt immunosuppressive treatment.

## Case report

A 55-year-old man with hypertension and a 20-pack-year smoking history presented with a two-day history of fever up to 39 °C and rapidly progressive dyspnea, preceded by several weeks of declining exercise tolerance. Past medical history was notable only for hypertension. He had no known autoimmune disease. Family history was negative for connective tissue diseases and interstitial lung disease. Social history revealed active smoking, alcohol social use, and occupational exposures (asbestos/silica/metal dust/birds/mold) were denied. There was no recent travel, sick contacts, or illicit drug use. No specific outpatient treatment was administered before hospital admission. On admission, he was tachypneic (respiratory rate, 35 cycles/min), hypoxemic (with SpO_2_ 80% of on room air, before oxygen supplementation), and in moderate respiratory distress. Physical examination revealed no skin rashes, mechanic’s hands, Gottron papules, heliotrope rash, Raynaud’s phenomenon, joint swelling or muscle weakness. Cardiovascular examination showed stable circulation without murmurs. Pulmonary auscultation revealed diminished breath sounds bilaterally with diffuse crackles. The abdomen was soft and nontender, with no peripheral edema. No neurological abnormalities were observed.

Laboratory tests demonstrated markedly elevated inflammatory markers: C-reactive protein (CRP) 490 mg/L, procalcitonin (PCT) 3.68 ng/mL and white blood cell count (WBC) 35 ×10^9^/L. Antigen tests for SARS-CoV-2, influenza A/B, respiratory syncytial virus (RSV), Mycoplasma pneumoniae and adenovirus were negative.

**Figure 1:**
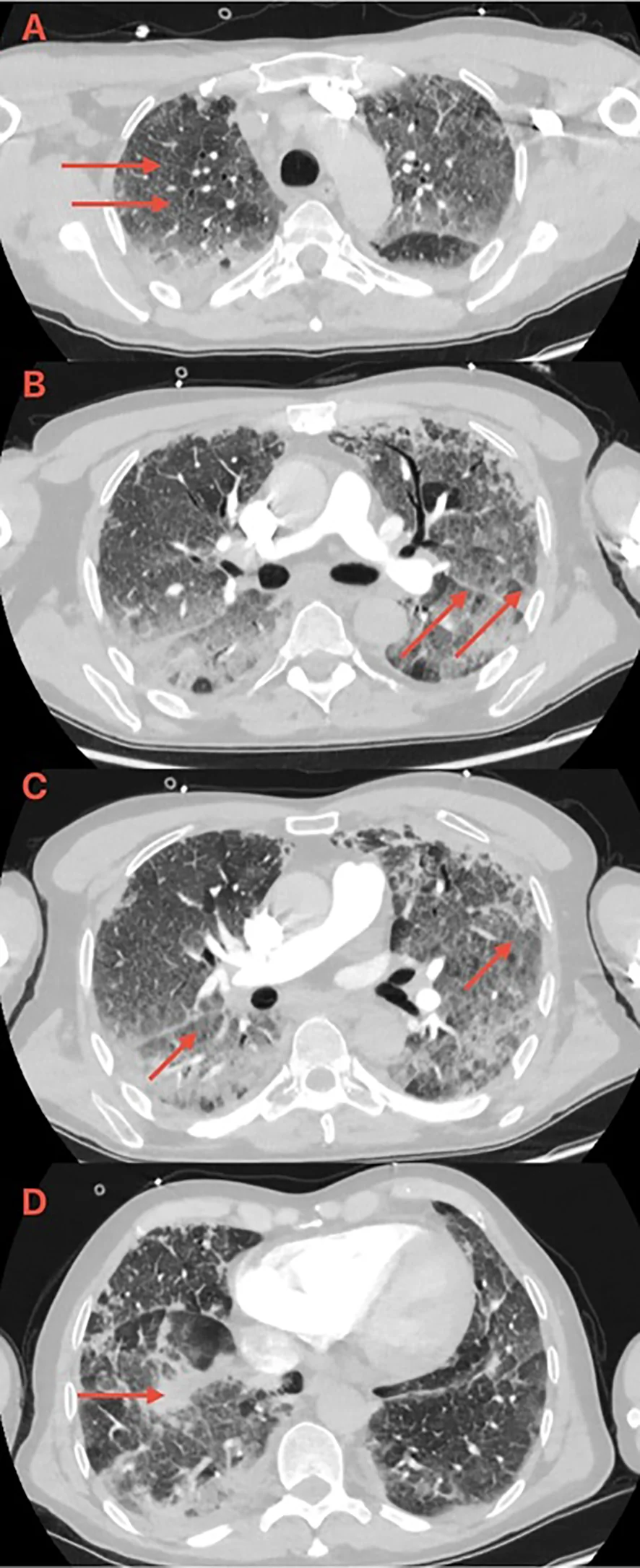
CT pulmonary angiogram on admission demonstrating features of the AIP pattern (red arrows): **(A)** nodular opacities. **(B)** interlobular septal thickening. **(C)** bilateral ground-glass opacities. **(D)** consolidations.

CT pulmonary angiogram excluded pulmonary embolism but revealed diffuse bilateral ground-glass opacities, interlobular septal thickening, numerous consolidations and nodular opacities, consistent with an AIP pattern (**Figure 1**).

Given the acute hypoxemic respiratory failure with diffuse bilateral opacities, the differential diagnosis at presentation included severe community-acquired pneumonia, noninfectious ARDS, acute exacerbation of an underlying ILD, diffuse alveolar hemorrhage, pulmonary vasculitis, drug-induced pneumonitis, acute eosinophilic pneumonia, and cardiogenic pulmonary edema. Pulmonary embolism was excluded by CT pulmonary angiogram. Empirical ceftriaxone and levofloxacin were initiated. Despite this, the patient’s respiratory status deteriorated, requiring high-flow nasal oxygen and subsequent endotracheal intubation. Arterial blood gas analysis while receiving HFNC (FiO_2_ 0.60) showed PaO_2_ 53.9 mmHg, corresponding to a PaO_2_/FiO_2_ ratio of 89.8 mmHg (severe hypoxemia in the severe ARDS range) (21). He developed hypotension requiring norepinephrine and acute kidney injury (AKI) requiring continuous renal replacement therapy (CRRT). Mechanical ventilation was challenging due to high inspiratory pressures and refractory hypoxemia, prompting prone positioning. Hemodynamic monitoring was performed using pulse index continuous cardiac output (PiCCO) and Swan–Ganz catheterization. Early bronchoscopy revealed no purulent secretions, and bronchial aspirate cultures remained sterile. Bronchoalveolar lavage was not performed due to the patient’s critical condition and severe hypoxemia; therefore, diffuse alveolar hemorrhage could not be definitively excluded by sequential BAL aliquot assessment. Transbronchial biopsy demonstrated acute interstitial inflammation compatible with the radiological impression of AIP (8, 10, 14, 22). Although inflammatory markers gradually decreased, respiratory failure persisted without clinical improvement.

### Diagnostic assessment

Diagnostic methods included serial physical examinations, laboratory testing, CT pulmonary angiogram, microbiological respiratory sampling, bronchoscopy without BAL (deferred due to severe hypoxemia), and transbronchial biopsy showing acute interstitial inflammation. Autoimmune testing included ANA by indirect immunofluorescence and a Myositis 3 immunoblot panel (EUROIMMUN). Diagnostic challenges were primarily related to clinical instability precluding BAL for definitive exclusion of diffuse alveolar hemorrhage and limiting invasive diagnostic procedures. Diagnostic reasoning evolved from presumed severe pneumonia to noninfectious acute lung injury due to persistent hypoxemia despite appropriate antimicrobials and sterile early cultures, an AIP-like imaging pattern, and subsequent identification of anti-EJ positivity supporting antisynthetase syndrome. Differential diagnoses considered included ARDS secondary to infection, acute exacerbation of underlying ILD, diffuse alveolar hemorrhage, pulmonary vasculitis, drug-induced pneumonitis, acute eosinophilic pneumonia, and cardiogenic pulmonary edema.”

After more than one week in the intensive care unit (ICU), repeat respiratory sampling grew *Acinetobacter baumannii*, consistent with ICU-acquired infection. Meropenem therapy was initiated. Despite appropriate microbiological coverage, the patient remained severely hypoxemic and ventilator-dependent, suggesting that infection alone could not explain the clinical course. Following discontinuation of sedation, the patient was awoken, oriented and in full verbal contact but exhibited profound generalized muscle weakness. Autoimmune serological testing revealed positive antinuclear antibodies (ANA) with a nucleolar (1:320), filamentous (1:320), speckled (1:160), and cytoplasmic (1:160) pattern, together with anti-EJ and anti-Th/To antibodies, supporting ASyS in the appropriate clinical context (7, 9). ANA was detected by indirect immunofluorescence (IIF). Myositis-specific/associated antibodies (including anti-EJ) were assessed using a commercial Myositis 3 immunoblot panel (EUROIMMUN, Lübeck, Germany). Creatine kinase (CK) at the time generalized weakness was recognized was [CK: 548 IU/L]; aldolase was not assessed. High-dose intreavenosus methylprednisolone (500 mg daily for three consecutive days), followed by oral prednisone at 1 mg/kg/day, was administered. Because of the lack of clinical improvement, intravenous cyclophosphamide was initiated (single dose of 1000 mg). Within 48 hours, the patient demonstrated marked improvement in oxygenation, hemodynamics and muscle strength. He was weaned from vasopressors, extubated via tracheostomy and subsequently decannulated. He continued to improve with rehabilitation.

**Figure 2:**
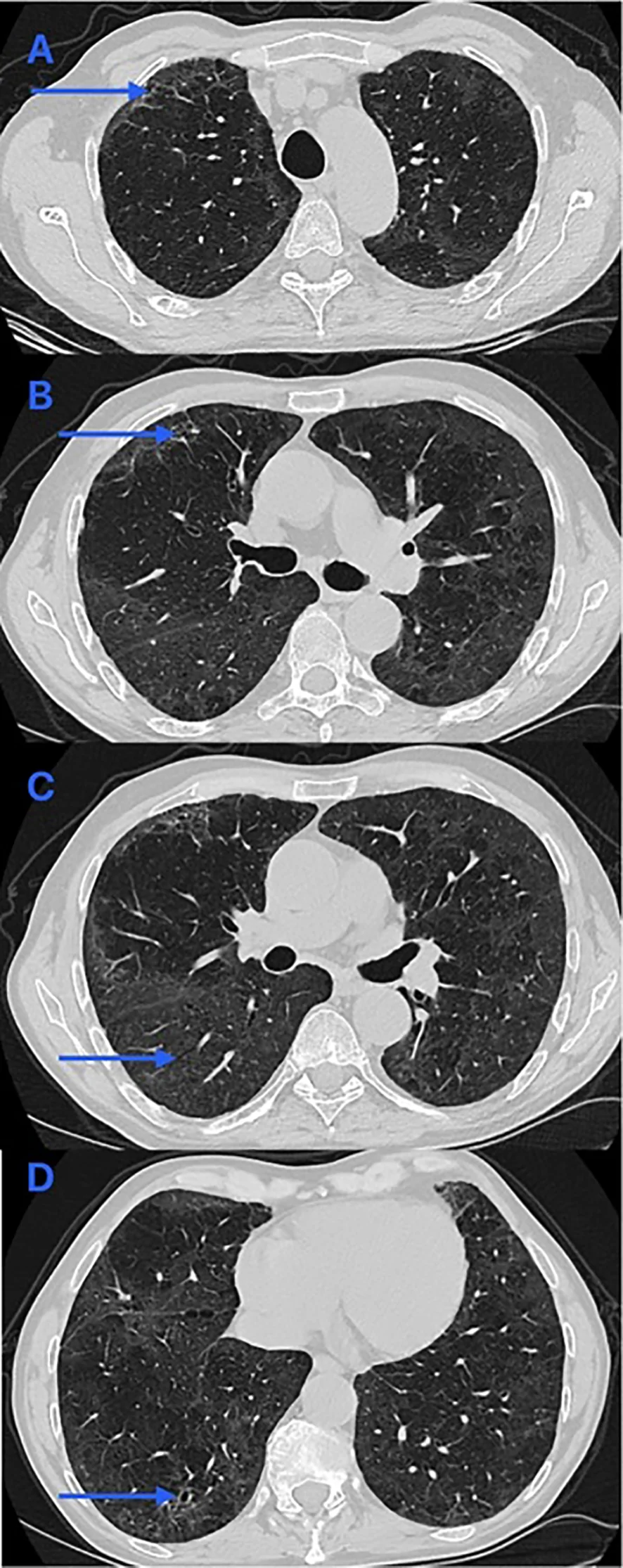
Follow-up chest CT at three weeks demonstrating significant regression of ground-glass opacities with (blue arrows): **(A, B)** limited honeycombing, consistent with NSIP/UIP overlap. **(C, D)** residual traction bronchiectasis.

Follow-up CT after three weeks demonstrated significant regression of ground-glass opacities and reticulation, with mild residual traction bronchiectasis and limited honeycombing, consistent with NSIP/UIP overlap pattern (**Figure 2**). A detailed timeline of the patient’s clinical course, diagnostic procedures, and treatment interventions is summarized in **Table 1**.

**Table 1:** Detailed study timeline with characteristics of patients condition including diagnostic and treatment procedures.

Time	Characteristics	
Day 0 Onset of symptoms	• Fever, cough, dyspnea	
Day 2	• Hospital admission	• Worsening of symptoms • Laboratory results: CRP 490 mg/dl, PCT 3,68 ng/mL, WBC 35 ×10^9^/L • AIP pattern on chest CT • Empirical antibiotic therapy • Respiratory deterioration
	• ICU admission	• Intubation under sedation • Vasopressor initiated due to hypotension • Intravenous GCS initiated
Day 8	• ICU hospitalization	• CRRT initiated due to progresive renal failure
Day 11		• Bronchoscopy with transbronchial biopsy
Weeks 2-3		• Tracheostomy • Sedation discontinued • Profound generalized muscle weakness
		• ANA (Th/To) and anti-EJ antibodies detected • AIP confirmed histopathologically • Antisynthetase syndrome suspected • High-dose corticosteroid therapy initiaded • First dose cyclophosphamide administered
		• Detection of *Acinetobacter baumanii* respiratory tract infection • Meropenem treatment • Improvement in oxygenation, hemodynamics and muscle strength • Weaned from vasopressors and mechanical ventilation; subsequently decannulated
Weeks 3-4	• Pulmonology department hospitalization	• Intravenous corticosteroids and antibiotic therapy continuation • Significant regression on chest CT • Clinical improvement and normalization of inflammatory markers

## Discussion

This case illustrates the diagnostic complexity of differentiating severe pneumonia from autoimmune ILD and emphasizes the need to consider noninfectious causes of antibiotic-refractory pneumonia. The initial presentation with fever, leukocytosis and diffuse infiltrates strongly suggested infection. However, the discordance between falling inflammatory markers and progressive respiratory failure is atypical for bacterial pneumonia (1, 4). The CT pulmonary angiogram demonstrated bilateral consolidations, these findings are nonspecific and may also occur in severe pneumonia, lung cancer or other causes of acute lung injury. In the present case, the lack of clinical improvement despite broad- spectrum antibiotic therapy, the sterile bronchoscopy and the absence of malignant cells in the trasbronchial biopsy supported a non-infectious, non-neoplastic ethiology and favored and underlying autoimmune process (8, 10, 14, 22). Although the patient fulfilled clinical criteria for ARDS (acute onset, bilateral opacities, severe hypoxemia), ARDS represents a syndrome rather than an etiology; in this case, the subsequent autoimmune findings supported an immunemediated cause of acute lung injury (21).

Anti-EJ ASyS is increasingly recognized as a distinct phenotype characterized by ILD-dominant disease, minimal or absent myositis and a high risk of RP-ILD (7–9, 11). Zhang et al. reported a cohort of 46 anti-EJ-positive patients, of whom 21.7% developed RP-ILD, confirming the aggressive pulmonary phenotype associated with this antibody specificity (11). In the present case, severe interstitial lesions were accompanied by profound muscle weakness that became apparent only after sedation withdrawal, highlighting that myositis may be masked in the ICU setting. Recent cohorts demonstrate that anti-EJ–positive patients frequently present with AIP-like patterns, severe hypoxemia and early respiratory failure, often requiring mechanical ventilation (7–9, 11). In the multicenter TYPASS cohort (n = 132), male sex, fever, and organizing pneumonia pattern were identified as independent risk factors for RP-ILD in ASyS, two of which were present in our patient (23). These findings were consistent with the clinical presentation of our patient.

Published case reports and small series of anti-EJ ASyS describe ILD-dominant presentations with acute/subacute deterioration that may mimic infection, with radiologic patterns ranging from NSIP/OP overlap to acute lung injury/DAD-like presentations. These reports support the clinical observation that anti-EJ positivity can be associated with severe early respiratory failure requiring intensive care and, in selected cases, rapid response after escalation to combination immunosuppression, including cyclophosphamide (7, 9, 24).

The mechanisms linking specific antisynthetase autoantibodies to an aggressive pulmonary phenotype remain incompletely defined. Proposed explanations include predominant lung-targeted immune activation in some non-Jo-1 antisynthetase subtypes and a propensity to develop acute lung injury patterns in susceptible hosts; however, current evidence is largely observational and hypothesis-generating (25).

In addition to the anti-ARS profile, extended autoimmunity testing in our patient identified anti-Th/To antibodies. Anti-Th/To is a recognized systemic sclerosis (SSc)–associated autoantibody and is classically linked to a nucleolar ANA pattern on HEp-2 indirect immunofluorescence; importantly, it has also been described in patients with minimal or absent skin involvement, including organ-dominant presentations such as ILD. Nevertheless, our patient currently lacks any clinical features of SSc; therefore, we interpret anti-Th/To as a serologic ‘red flag’ warranting increased diagnostic vigilance rather than a stand-alone basis to revise the working diagnosis. From a practical standpoint, this serologic finding supports targeted surveillance for cardiopulmonary complications—particularly pulmonary hypertension (PH), which occurred in **38%** of anti-Th/To–positive SSc patients during long-term follow-up in a large case–control cohort (**n=204**)—and it also justifies assessment for SSc-type microangiopathy (e.g., nailfold capillaroscopy) when SSc is considered (26–28).

Early immunosuppression is critical in RP-ILD associated with ASyS. The 2023 American College of Rheumatology/American College of Chest Physicians (ACR/CHEST) guideline for treatment of ILD in systemic autoimmune rheumatic diseases conditionally recommends cyclophosphamide over mycophenolate mofetil and azathioprine for RP-ILD, with intravenous administration generally preferred over oral therapy (29). Cyclophosphamide provides rapid and profound immune suppression, with evidence showing improvement in forced vital capacity, diffusing capacity, and chest CT findings in 67–71% of patients with IIM-associated ILD (30, 31). Rituximab represents an alternative, with the RECITAL trial demonstrating similar efficacy to cyclophosphamide in connective tissue disease-associated ILD (32). Emerging data suggest potential benefit of Janus kinase (JAK) inhibitors in refractory cases (33). In our patient, the rapid improvement after escalation of immunosuppression was clinically consistent with an inflammatory/immune-mediated process, although causality cannot be definitively established from a single case.

In critically ill patients with RP-ILD and respiratory failure, ACR/CHEST emphasizes IV pulse methylprednisolone due to rapid onset and supports early combination therapy with an additional immunosuppressive agent; cyclophosphamide is conditionally preferred over mycophenolate and azathioprine in this emergency phenotype, while rituximab is an alternative option. In our patient, the immediacy and severity of deterioration (mechanical ventilation, vasopressors, CRRT) favored an induction regimen with IV glucocorticoids plus IV cyclophosphamide to achieve rapid disease control (29).

Although rare, DAH has been reported in ASyS and other IIM (15–17). Awareness of this possibility is important, particularly in patients with diffuse ground-glass opacities and acute respiratory failure. In the present case, the absence of hemoptysis and the response to immunosuppression favored AIP-like pattern lung injury over DAH; however, BAL with sequential aliquot analysis was not performed due to clinical instability, which remains a limitation. The diagnostic certainty in this case is strengthened by applying the2024 Classification Criteria for Anti-Synthetase Syndrome (CLASS), which integrate serologic markers with weighted clinical and organ-specific manifestations (19, 20). The CLASS criteria recognize that ASyS can present with ILD as the dominant or sole manifestation, particularly in anti-EJ-positive patients, and that myositis may be absent or develop later in the disease course (20, 25). Earlier systems, including the 2017 EULAR/ACR IIM criteria, did not classify ASyS as a separate entity (19).

Strengths of this report include detailed imaging evolution with short-interval follow-up and comprehensive serological assessment supporting anti-EJ ASyS. Limitations include the absence of BAL (and therefore inability to definitively exclude DAH), reliance on transbronchial biopsy rather than surgical lung biopsy to characterize diffuse alveolar damage, and the inherent limitation of causal inference from a single case, particularly in the setting of critical illness and ICU-acquired infection.

### Patient perspective

After recovery, the patient reported that the abrupt onset of breathlessness and ICU course were distressing and that improvement after immunosuppressive therapy was perceived as rapid and meaningful. He expressed understanding of the need for long-term follow-up for ILD and potential adverse effects of immunosuppression.

## Conclusions

ASyS should be considered in severe pneumonia-like presentations that are unresponsive to antibiotics therapy, particularly in patients with rapidly progressive ILD (1, 4, 7–9, 11). Anti-EJ antibodies are associated with AIP-like radiological patterns and fulminant respiratory failure (7–9). Early serologic testing and prompt immunosuppression can be life-saving. Radiologic evaluation, bronchoscopy and comprehensive autoantibody testing are essential for diagnosis. Awareness of uncommon pulmonary manifestations such as DAH is increasingly important (15–17). The recently published 2024 Classification Criteria for Anti-Synthetase Syndrome (CLASS)provide a modern framework for accurate classification of ASyS, especially in ILD-dominant presentations (19, 20).

## Data Availability

The raw data supporting the conclusions of this article will be made available by the authors, without undue reservation.
